# Supplementary figures and images for: Experimentally induced pain does not influence updating of peripersonal space and body representations following tool-use
Source: PLoS One. 2019 May 16;14(5):e0210045. doi: 10.1371/journal.pone.0210045 (PMC6522125; doi:10.1371/journal.pone.0210045)

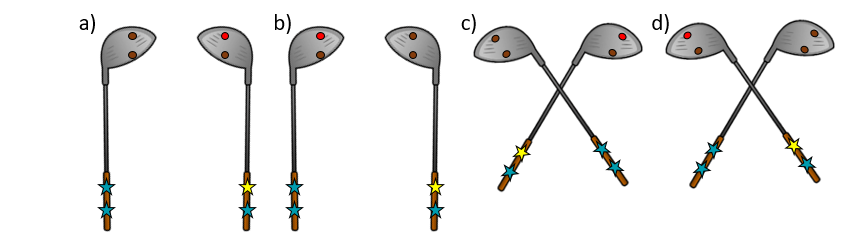

Supplement: S1 Fig — The different possible combinations for the Crossmodal Congruency task (CCT) for congruent visuotactile stimulation. The brighter red dots illustrate the location of the visual stimulation, and the yellow stars the location of the tactile stimulation for each example trial. The tools could be in the straight (A & B), or crossed (C & D) position, with light appearing in the same (A & C) or opposite (B & D) visual field as the visuotactile stimulation. (TIF) [file pone.0210045.s001.tif]

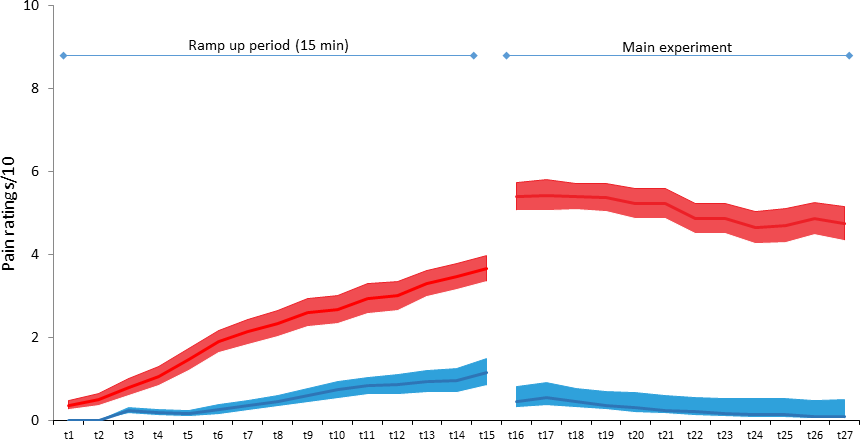

Supplement: S2 Fig — Change in pain ratings over time in the pain (red line) and active placebo (blue line) conditions. The neutral condition was omitted as mean ratings were ≈ 0. The ramp up period (t1-t15) lasted 15 min for the active placebo and neutral conditions. For the pain condition, this period lasted until pain ratings reached 5/10, or plateaued at 3/10 or higher for 3 consecutive ratings, which took on average 16.7 minutes (SD = 1.88). Pain ratings were recorded every minute during the ramp up period. When the ramp up period was shorter than 15 minutes, the missing ratings were adjusted to 5/10 for the purpose of this figure. Pain ratings for ramp up periods exceeding 15 minutes are not included in this figure. During the main experiment (t16-t27) pain ratings were recorded between the tactile distance judgements and different set of the tool-use tasks, and so the time between pain ratings was variable. (TIF) [file pone.0210045.s002.tif]

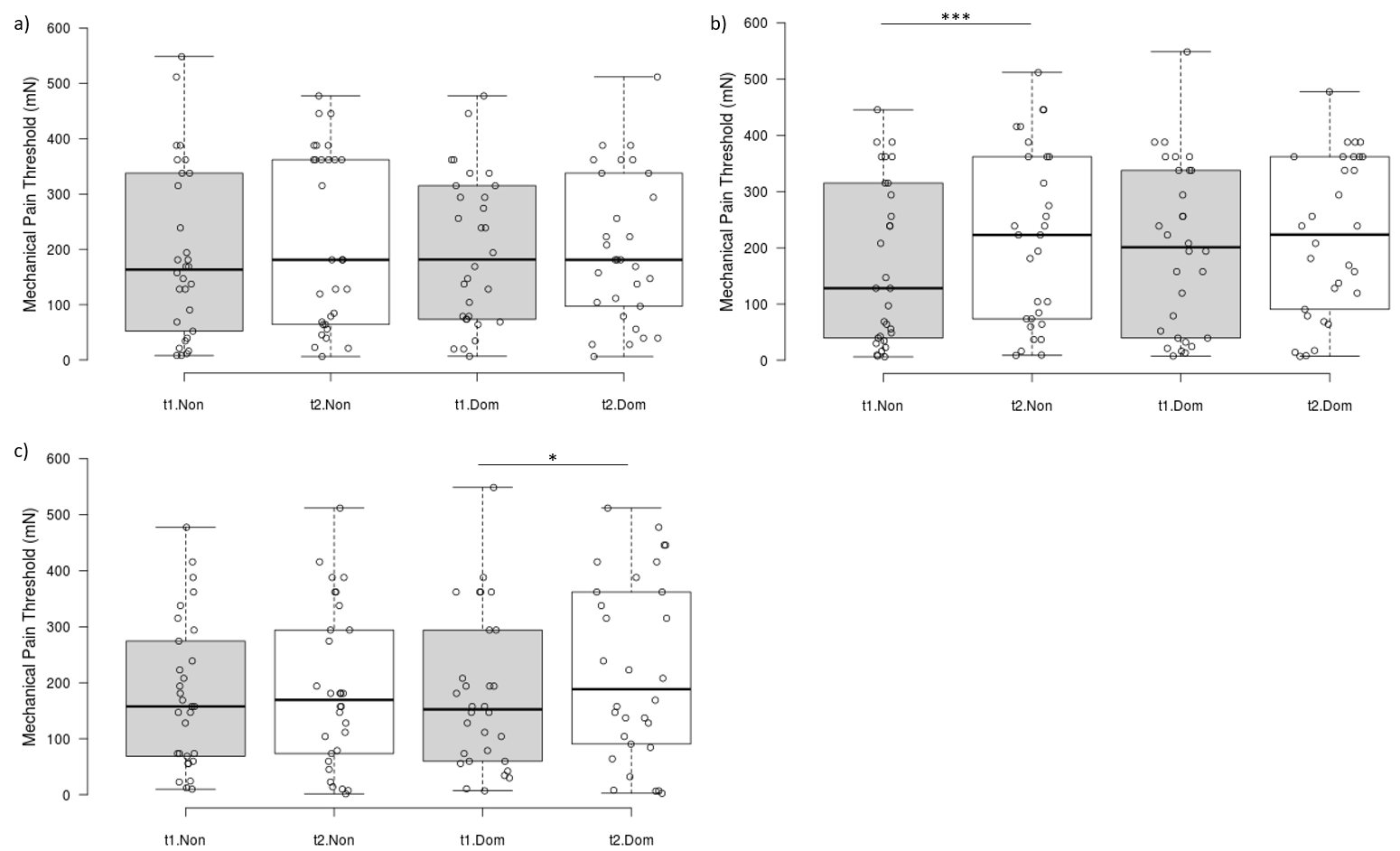

Supplement: S3 Fig — Mechanical Pain Threshold (MPT) in mN on the middle finger (D3) before and after pain induction, active placebo, or no sensory manipulation (neutral), split by Side of Body for each conditions: neutral (A), active placebo (B), and pain (C). Circles depict individual data points. The dominant arm was always stimulated. (N = 30), * p < .05, ** p < .01. (TIF) [file pone.0210045.s003.tif]

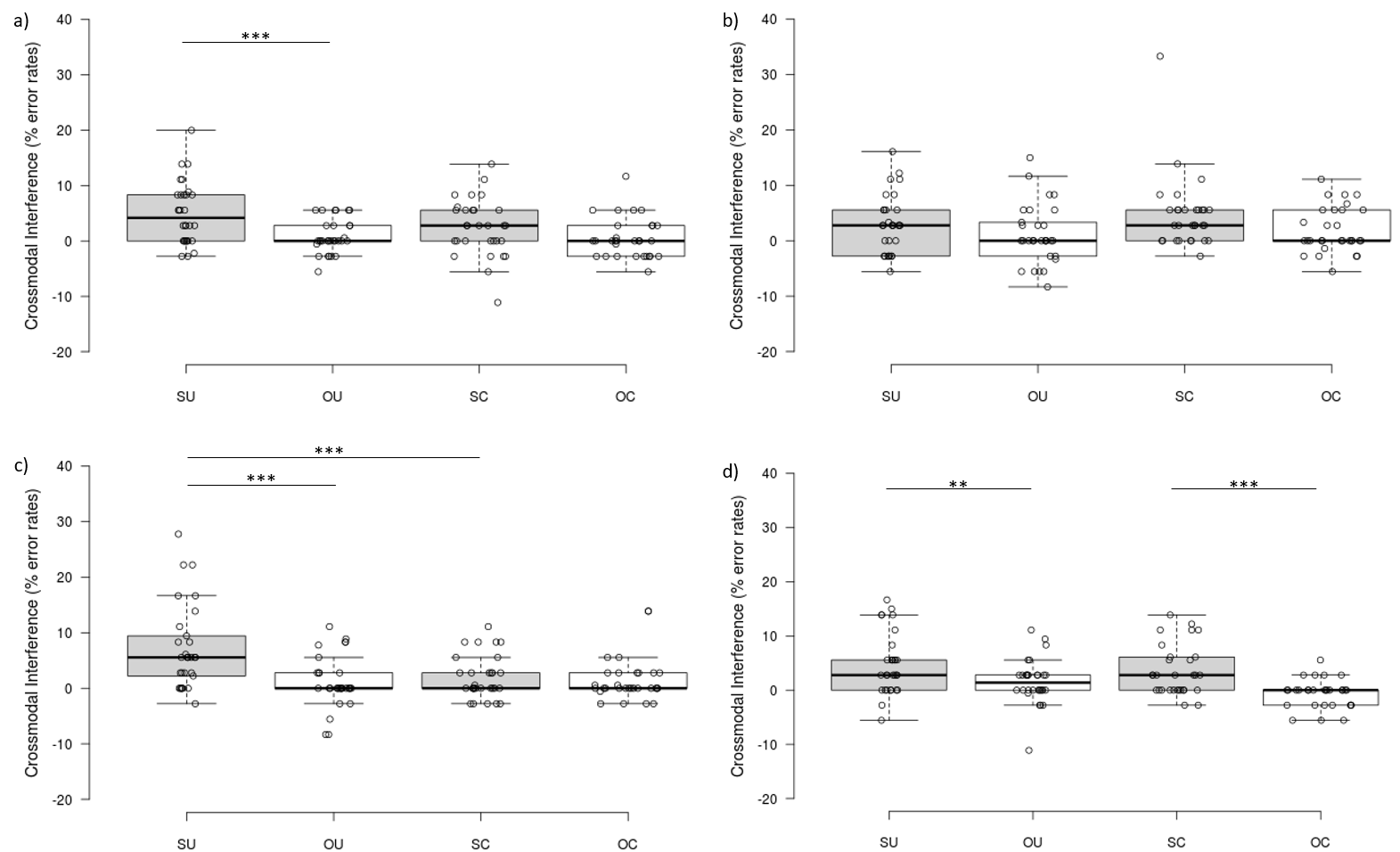

Supplement: S4 Fig — Crossmodal interference shown by Set (1 [passive], 2, 3, 4), Tool Arrangement (uncrossed, crossed), and Visual Field (same, opposite) for error rates expressed in percentages on the Crossmodal Congruency Task (CCT), for all participants (n = 30). Crossmodal interference was calculated by subtracting congruent from incongruent error rates. Medians are depicted by the centre lines. The box limits indicate the 25ht and 75th percentile. The whiskers extend 1.5 times the interquartile range from the box limits. Circles depict individual data points. (N = 30). ** p < .010, *** p < .001. (TIF) [file pone.0210045.s004.tif]
