# Supplementary material for: Experimentally induced pain does not influence updating of peripersonal space and body representations following tool-use
Source: PLoS One. 2019 May 16;14(5):e0210045. doi: 10.1371/journal.pone.0210045 (PMC6522125; doi:10.1371/journal.pone.0210045)
Supplement: S1 Table — Performance on sensory tests (Mechanical pain Threshold [MPT], Mechanical Detection Threshold [MDT], Two Point Discrimination [TPD]) expressed as change from pre to post Sensory Manipulation. Means and standard deviations for Sensory tests are split by Sensory Condition (Pain, Active Placebo, Natural), and Side of Body (dominant [stimulated], non-dominant). (DOCX) [file pone.0210045.s005.docx]

|  | **MPT (mN)** | | **MDT (g)** | | **TPD (mm)** | |
| --- | --- | --- | --- | --- | --- | --- |
|  | ***M*** | **SD** | ***M*** | **SD** | ***M*** | **SD** |
| Dominant |  |  |  |  |  |  |
| Pain | 46.32 | 108.48 | 0.00 | 0.01 | 0.19 | 0.82 |
| Active Placebo | 18.58 | 61.03 | 0.00 | 0.01 | -0.02 | 0.44 |
| Neutral | 1.62 | 48.95 | 0.00 | 0.01 | 0.04 | 0.35 |
| Non-Dominant |  |  |  |  |  |  |
| Pain | 10.88 | 100.21 | 0.00 | 0.01 | -0.13 | 0.47 |
| Active Placebo | 48.89 | 70.65 | 0.00 | 0.01 | -0.06 | 0.37 |
| Neutral | 25.27 | 86.15 | 0.00 | 0.01 | 0.06 | 0.39 |

**S1 Table. Performance on sensory tests.** Performance on sensory tests (Mechanical Pain Threshold [MPT], Mechanical Detection Threshold [MDT], Two Point Discrimination [TPD]) expressed as change from pre to post Sensory Manipulation. Means and standard deviations for Sensory tests are split by Sensory Condition (Pain, Active Placebo, Natural), and Side of Body (dominant [stimulated], non-dominant).
