# Supplementary material for: Experimentally induced pain does not influence updating of peripersonal space and body representations following tool-use
Source: PLoS One. 2019 May 16;14(5):e0210045. doi: 10.1371/journal.pone.0210045 (PMC6522125; doi:10.1371/journal.pone.0210045)
Supplement: S2 Table — All significant main effects interactions from six-way ANOVA of Sensory Condition, Side of Body, Set, Tool Arrangement, Visual Field, and Congruence from the Crossmodal Congruency Task for reaction times. (DOCX) [file pone.0210045.s006.docx]

|  | ***F*** | ***p*** | **ƞ^2^_p_** |
| --- | --- | --- | --- |
| Main effects |  |  |  |
| Set | 46.43 | <.001 | .62 |
| Side of Body | 7.97 | .009 | .22 |
| Visual Field | 6.69 | .015 | .19 |
| Congruence | 177.19 | <.001 | .86 |
| Two-way |  |  |  |
| Set x Side of Body | 3.19 | .039 | .26 |
| Tool Arrangement x Visual Field | 10.56 | .003 | .27 |
| Visual Field x Congruence |  |  |  |
| Three-way |  |  |  |
| Sensory Condition x Tool Arrangement x Visual Field | 3.39 | .048 | .20 |
| Side of Body x Tool Arrangement x Visual Field | 15.5 | <.001 | .35 |
| Set x Tool Arrangement x Congruence | 4.36 | .013 | .33 |
| Tool Arrangement x Visual Field x Congruence | 9.43 | .005 | .25 |
| Four-way |  |  |  |
| Set x Tool Arrangement x Visual Field x Congruence | 3.28 | .035 | .10 |

**S2 Table. CCT main effects – reaction times**. All significant main effects interactions from six-way ANOVA of Sensory Condition, Side of Body, Set, Tool Arrangement, Visual Field, and Congruence from the Crossmodal Congruency Task for reaction times.
