# Supplementary material for: Experimentally induced pain does not influence updating of peripersonal space and body representations following tool-use
Source: PLoS One. 2019 May 16;14(5):e0210045. doi: 10.1371/journal.pone.0210045 (PMC6522125; doi:10.1371/journal.pone.0210045)
Supplement: S3 Table — Descriptive statistics presented for the reaction times in ms from the crossmodal congruency task, split by Tool Arrangement (uncrossed, crossed), Side of Body (same, opposite), and Congruence (congruent, incongruent). The difference between congruent and incongruent scores are reported within each level of Tool Arrangement, and Side of Body. (DOCX) [file pone.0210045.s007.docx]

|  | **Congruent** | | **Incongruent** | | ***t*** | ***p*_adjusted_** |
| --- | --- | --- | --- | --- | --- | --- |
|  | ***M*** | ***SD*** | ***M*** | ***SD*** |  |  |
| Uncrossed |  |  |  |  |  |  |
| Same | 630.11 | 89.92 | 702.85 | 93.04 | 13.45 | .004 |
| Opposite | 664.73 | 93.63 | 691.89 | 95.26 | 5.29 | .004 |
| Crossed |  |  |  |  |  |  |
| Same | 641.03 | 99.28 | 695.21 | 99.63 | 8.47 | .004 |
| Opposite | 651.73 | 91.50 | 683.07 | 98.30 | 7.91 | .004 |

**S3 Table. CCT reaction times**. Descriptive statistics presented for the reaction times in ms from the crossmodal congruency task, split by Tool Arrangement (uncrossed, crossed), Side of Body (same, opposite), and Congruence (congruent, incongruent). The difference between congruent and incongruent scores are reported within each level of Tool Arrangement, and Side of Body.
