# Supplementary material for: Experimentally induced pain does not influence updating of peripersonal space and body representations following tool-use
Source: PLoS One. 2019 May 16;14(5):e0210045. doi: 10.1371/journal.pone.0210045 (PMC6522125; doi:10.1371/journal.pone.0210045)
Supplement: S4 Table — All significant main effects interactions from six-way ANOVA of Sensory Condition, Side of Body, Set, Tool Arrangement, Visual Field, and Congruence from the Crossmodal congruency task for error rates. (DOCX) [file pone.0210045.s008.docx]

|  | ***F*** | ***p*** | **ƞ^2^_p_** |
| --- | --- | --- | --- |
| Main effects |  |  |  |
| Side of Body | 7.38 | .011 | .20 |
| Tool Arrangement | 8.30 | .007 | .22 |
| Visual Field | 22.05 | <.001 | .43 |
| Congruence | 38.88 | <.001 | .57 |
| Two-way |  |  |  |
| Side of Body x Tool Arrangement | 4.26 | .048 | .13 |
| Side of Body x Congruence | 7.12 | .012 | .20 |
| Visual Field x Congruence | 47.14 | <.001 | .62 |
| Three-way |  |  |  |
| Side of Body x Tool Arrangement x Visual Field | 4.17 | .050 | .13 |
| Four-way |  |  |  |
| Set x Tool Arrangement x Visual Field x Congruence | 4.47 | .011 | .33 |
| Six-way |  |  |  |
| Sensory Condition x Side of Body x Set x Tool Arrangement x Visual Field x Congruence | 2.73 | .031 | .09 |

**S4 Table. CCT main effects – error rates**. All significant main effects interactions from six-way ANOVA of Sensory Condition, Side of Body, Set, Tool Arrangement, Visual Field, and Congruence from the Crossmodal congruency task for error rates.
