# Supplementary material for: Experimentally induced pain does not influence updating of peripersonal space and body representations following tool-use
Source: PLoS One. 2019 May 16;14(5):e0210045. doi: 10.1371/journal.pone.0210045 (PMC6522125; doi:10.1371/journal.pone.0210045)
Supplement: S1 Text — (DOCX) [file pone.0210045.s009.docx]

**S1 Text. Crossmodal congruency task (CCT) results – error rates**

All significant main effects and interactions are reported in S4 Table. There were main effects of Side of Body, *F*(2, 29) = 7.38, *p* = .011, ƞ^2^_p_ = .20, Tool Arrangement, *F*(1, 29) = 8.30, *p* = .007, ƞ^2^_p_ = .22, Visual Field, *F*(1, 29) = 22.05, *p* < .001, ƞ^2^_p_ = .43, and Congruence, *F*(1, 29) = 38.88, *p* < .001, ƞ^2^_p_ = .57, on error rates from the CCT. Error rates were higher for uncrossed (*M* = 3.47 %, *SD* = 2.19) than for crossed (*M* = 2.71 %, *SD* = 1.64) tools. More errors were made when visual distractors appeared in the same (*M* = 3.65 %, *SD* = 2.74) than the opposite (*M* = 2.53 %, *SD* = 1.64) visual field relative to vibrotactile targets. Participants had higher error rates for trials where the vertical locations of visual and vibrotactile stimulation were incongruent (*M* = 4.35 %, *SD* = 3.29), compared to congruent (*M* = 1.84 %, *SD* = 1.10).

The Tool Arrangement x Visual Field x Congruence interaction (Fig 3) was not significant for error rates from the CCT, *F*(1, 29) = 3.68, *p* = .065, ƞ^2^_p_ = .13. There was a four-way interaction (S4 Fig) between Set, Tool Arrangement, Visual Field, and Congruence on error rates, *F*(2, 28) = 4.47, *p* = .011, ƞ^2^_p_ = .33. Follow-up ANOVAs revealed a significant interaction between Tool Arrangement, Visual Field, and Congruence at set 3, *F*(1, 29) = 13.28, *p* = .001, ƞ^2^_p_ = .31, but not in any other sets, *Fs*(1, 29) ≤ 1.68, *ps* ≥ .183, ƞ^2^_ps_ ≤ .06. To further investigate these patterns of results we calculated the crossmodal interference by subtracting error rates for the congruent condition from error rates for the incongruent condition, and compared this across each level of Tool Arrangement and Visual Field with each set. Follow-up tests for set 3 showed that crossmodal interference was higher for same (*M* = 7.07, *SD* = 7.55) than opposite (*M* = 1.11, *SD* = 4.59) visual field distractors relative to the vibrotactile stimulation when the tools were uncrossed, *t*(29) = 4.78, *p*_adjusted_ = .004, *d* = 1.78. For distractors appearing in the same visual field, crossmodal interference from set 3 was higher for uncrossed than crossed (*M* = 1.96, *SD* = 4.59) tools, *t*(29) = 3.94, *p*_adjusted_ = .009, *d* = 1.46. No other follow-up comparisons from set 3 were significant, *t*s(29) ≤ 0.73, *ps*_adjusted_ ≥ .941, *ds* ≤ 0.27. The three-way interaction in set 3 for error rates from the CCT appears to be driven by greater crossmodal interference for visual distractors in the same compared to opposite visual field as tactile targets, when tools were uncrossed, but not crossed. This is consistent with a remapping of peripersonal space representations to accommodate tool-use. These findings indicate that changes in peripersonal space may have been observed in set 3. We did not observe this pattern for any other sets, as the three-way interaction Tool Arrangement x Visual Field x Congruence was only present during set 3.

There was a six way interaction between Sensory Condition, Set, Side of Body, Tool Arrangement, Visual Field, and Congruence in the analysis of error rates, *F*(4.14, 120.08) = 2.73, *p* = .031, ƞ^2^_p_ = .09. Upon further analysis, however, there were no effects that were clearly driven by Sensory Condition from this interaction. We conducted four separate Set x Tool Arrangement x Visual Field x Congruence ANOVAs on the data split by Sensory Condition and Side of Body. A three-way interaction between Tool arrangement, Visual Field, and Congruence was present for error rates for tactile stimulation delivered to the non-dominant arm in the pain condition, *F*(1, 29) = 5.31, *p* = .029, ƞ^2^_p_ = .16, but not in any of the other Sensory Condition by Body Side conditions, *F*s ≤ 1.19, *p*s ≥ .284 , ƞ^2^_p_s. ≤ .04. Follow-up t-tests revealed that these results were driven by greater crossmodal interference for distractors in the same (*M* = 5.78 %, *SD* = 7.99) than the opposite (*M* = 0.28 %, *SD* = 5.57) visual field relative to the target location for uncrossed tools, *t*(29) = 2.88, *p*_adjusted_ = .016, *d* = 1.07. However, no other follow-up tests were significant for the non-dominant arm in the pain condition, *t*s(29) ≤ 1.76, *ps*_adjusted_ ≥ .270, *ds* ≤ 0.65. This result is consistent with peripersonal space representations being updated to accommodate the tool.

There was a four-way interaction of Set, Tool Arrangement, Visual Field, and Congruence for tactile stimulation delivered to the dominant hand in the neutral condition, *F*(3, 27) = 3.99, *p* = .018, ƞ^2^_p_ = .31. Follow-up tests revealed that this was driven by significant Tool Arrangement x Visual Field x Congruence interaction in set 1 (passive), *F*(1, 29) = 10.25, *p* = .003, ƞ^2^_p_ = .26, and in set 4, *F*(1, 29) = 4.96, *p* = .034, ƞ^2^_p_ = .15, but not in sets 2 and 3, *F*s≤ 1.44, *p*s ≥ .240, ƞ^2^_p_ ≤ .05. For the dominant hand in the neutral condition, there was greater crossmodal interference for visual distractors appearing in the same (*M* = 8.56, *SD* = 11.57) than opposite (*M* = -0.56, *SD* = 8.45) visual field relative to vibrotactile targets, for uncrossed tools in set 1, *t*(29) = 3.13, *p*_adjusted_ = .006, *d* = 1.16. Crossmodal interference was greater for uncrossed than crossed (*M* = -1.11, *SD* = 10.66) tools when visual distractors appeared in the same visual field relative to vibrotactile targets, for the dominant hand in the neutral condition for set 1, *t*(29) = 3.13, *p*_adjusted_ = .030, *d* = 1.26. No other follow-up comparisons for the dominant hand in the neutral condition set 1 were significant, *t*s(29) ≤ 0.72, *ps*_adjusted_ ≥ .994, *ds* ≤ 0.27. No follow-up test for the dominant hand in the neutral condition in set 4 withstood correction for multiple comparisons, *t*s(29) ≤ 3.10, *ps*_adjusted_ ≥ .104, *ds* ≤ 1.15. This pattern indicates that peripersonal space representations were updated for set 1 only, for the dominant hand in the neutral condition. Therefore, there was no evidence of a change in the overall pattern of accuracy over time that would indicate emergence of updating of peripersonal space.

Our results suggest that peripersonal space extended to include the tips of the tools from as early as set 1, in which participants interacted only passively with the tools. This observation is contrary to previous findings in which extension of peripersonal space only occurred after a period of active tool use, and emerged over time with on-going tool use.

**Exploratory analyses**

We considered that experience with the tool in session one might have primed participants to rapidly embody the tools upon grasping the handles of the tools at the beginning of sessions 2 and 3, and extend peripersonal space even while passively interacting with them. Because the order of the study was randomised and counterbalanced, we could investigate this possibility by conducting a between groups analysis of the data from the first session. Furthermore, there were six possible orders in which participants could have completed the study. We could use this information to create a categorical variable to enable us to further explore any order effects. Thus, we conducted two five-way ANOVAs on the RTs and error rates from the first experimental session with Set, Side of Body, Tool Arrangement, Visual Field and Congruence as within-subjects factors; and Sensory Condition as a between-subjects factor with ten participants in each of the pain, active placebo, and neutral groups. There was no main effects of Sensory Condition error rates from the CCT, *F*(2, 52) = 1.00, *p* = .390, ƞ^2^_p_ = .07. There was an interaction between Sensory Condition, Set, Side of Body, Tool Arrangement, Visual Field, and Congruence on error rates, *F*(4.74, 64.05) = 3.17, *p* = .014, ƞ^2^_p_ = .19. We followed this analysis up with six ANOVAs of the effects of Set, Tool Arrangement, Visual Field, and Congruence on error rates, within each level of Sensory Condition and Side of Body. However, none of the follow-up ANOVAS showed a significant interaction between Tool Arrangement, Visual Field, and Congruence, *Fs*(1, 9) ≤ 2.82, *ps* ≥ .128, ƞ^2^_p_s ≤ .24.
